# Coronavirus Disease 2019 and Takotsubo Syndrome

**DOI:** 10.31083/j.rcm2309298

**Published:** 2022-09-05

**Authors:** Ling Zhou, Zijun Chen, Riyue Jiang, Yimin Hu, Bin Zhu, Chun Yang, Ling Yang, Cunming Liu

**Affiliations:** ^1^Department of Anesthesiology and Perioperative Medicine, The First Affiliated Hospital of Nanjing Medical University, 210029 Nanjing, Jiangsu, China; ^2^Department of Cardiology, The Third Affiliated Hospital of Soochow University, 213003 Changzhou, Jiangsu, China; ^3^Department of Ultrasound Imaging, Renmin Hospital, Wuhan University, 430060 Wuhan, Hubei, China; ^4^Department of Anesthesiology, The Second Affiliated Changzhou People's Hospital of Nanjing Medical University, 213000 Changzhou, Jiangsu, China; ^5^Department of Critical Care Medicine, The Third Affiliated Hospital of Soochow University, 213003 Changzhou, Jiangsu, China

**Keywords:** COVID-19, diagnosis, pathogenesis, Takotsubo syndrome, treatment

## Abstract

The novel coronavirus disease 2019 (COVID-19) pandemic caused by severe acute respiratory syndrome coronavirus 2 (SARS-CoV-2) has become a global public health emergency. As the number of confirmed cases increases, cardiovascular complications, such as myocardial injury and cardiac dysfunction, are evidenced. Takotsubo syndrome (TTS), which is common in the intensive care unit, is diagnosed among COVID-19 patients. There have been 68 more cases reports with over 119 patients since a COVID-19 patient with TTS was first reported on April 14, 2020. Angiotensin-converting enzyme 2 (ACE2), which is widely expressed in the lungs and heart, is the virus receptor. Nevertheless, randomized studies on COVID-19 related TTS are lacking, and the pathogenesis and pathophysiology are still unclear. Therefore, this review provides an overview of the potential pathogenesis, pathophysiology, clinical manifestations, diagnosis, and treatment strategy for TTS in the COVID-19 era based on current practices.

## 1. Introduction

Coronavirus disease 2019 (COVID-19) caused by severe acute respiratory syndrome coronavirus 2 (SARS-CoV-2) has become a global public health issue and has affected more than 200 countries worldwide. As of May 8, 2022, the World Health Organization had announced more than 513 million confirmed cases and more than 6,249,700 global deaths, and the number is still rising.

It has been reported that SARS-CoV-2 is more contagious than SARS-CoV and the Middle East respiratory syndrome coronavirus in 2003 and 2015, respectively. However, the mechanism of SARS-CoV-2 infection is similar to that of the traditional coronaviruses, that is, through angiotensin-converting enzyme 2 (ACE2) receptors, which is widely expressed in the lungs, heart, kidneys, and gut [1]. The localization of receptors correlates with presenting symptoms and organ dysfunction [2]. Recently, myocardial injury was observed among COVID-19 patients with a morbidity rate of 19.7%, and patients with cardiac injury exhibited a higher mortality rate of 51.2% [3]. There have been 68 cases reports with similar clinical symptoms in the COVID-19 era since a case of typical Takotsubo syndrome (TTS) triggered by SARS-CoV-2 was first reported on April 14, 2020 [4] (**Supplementary Material 1**). Acute onset of TTS in hospitalized patients is not uncommon, and more importantly, the risk of overall mortality was not trivial [5]. Awareness of the need to distinguish TTS in the COVID-19 era is exceedingly necessary. This review outlines the epidemiology, manifestations, pathogenesis, and therapeutic strategies of TTS triggered by the COVID-19 pandemic.

## 2. Takotsubo Syndrome

TTS is characterized by transient and reversible left ventricular (LV) systolic dysfunction, which is frequently preceded by emotional or physiological stress [6]. The term “Takotsubo” is derived from the specific appearance of a resemblance of an octopus pot at the LV end-diastolic phase. TTS mimics the clinical manifestations in patients with acute coronary syndrome (ACS) (e.g., acute chest pain, T-wave inversion, and ST-segment elevation) and must be differentiated from it [6]. Notably, TTS is not an acute myocardial injury caused by coronary artery stenosis or obstruction. However, its serious cardiac complications are similar to ACS, which increasingly receives more attention [6].

### 2.1 TTS Epidemiology

Since the first report of TTS in 1991, its prevalence has indicated an increasing trend in the past decades [7]. In relation to the increased awareness of TTS, the incidence of TTS increased from 315 cases in 2006 to 6230 cases in 2012 [8]. TTS could now be easily differentiated from ACS owing to the advancement of efficient diagnostic techniques such as invasive coronary angiography. The prevalence of TTS was reported to be approximately 2% in patients with suspected ACS (up to 10% in women), and 85%–90% of TTS patients are postmenopausal women [6, 9]. Nevertheless, the prevalence of TTS is still underestimated because of immature diagnostic criteria, such as the possibility of coexisting coronary artery disease (CAD).

### 2.2 Predisposing Factors of TTS 

TTS is often triggered by strong mental or physical stressors and has been described as ‘stress cardiomyopathy’. Both negative and positive emotions can trigger TTS [6, 10]. Males tend to be more affected by physical stressors, whereas women are more susceptible to emotional stressors [11]. Physical stressors include severe somatic diseases, such as acute respiratory failure, severe infection, and malignant tumors [12]. In addition, surgery, invasive tests, and exogenous drugs, such as glucocorticoids and catecholamines, may act as physical stressors for the onset of TTS [12].

Although few people develop TTS, acute psychological and physiological stressors are universal, which predispose individuals to be more susceptible to TTS. Abnormality in the autonomic nervous system and catecholamine sensitivity caused by mental and nervous system disorders (stroke, subarachnoid occurrence, epilepsy, etc.) and specific sensitive adrenergic receptor genotypes increase TTS susceptibility [13, 14, 15]. Moreover, special cardiac anatomical structures, such as dysplasia of the hypoplastic branches of the apical coronary arteries and LV outlet tract obstruction, may act as risk factors of TTS [16, 17]. Interestingly, the cardioprotective effect of estrogen weakens after menopause, potentially causing a higher incidence of the onset of TTS [18].

### 2.3 Pathogenesis of TTS

Although the exact pathogenesis of TTS is unclear, there is considerable evidence that sympathetic activation might play a key role. In addition, markedly elevated levels of catecholamines may contribute to the onset of TTS [19, 20]. The distribution of cardiac sympathetic nerve endings was gradually decreased from the basal ventricular myocardium to the apical myocardium. The density and sensitivity of β-adrenergic receptors (βARs) were increased from the base to the apex to compensate for this gradient [21, 22]. At physiological concentrations, catecholamines stimulate a positively inotropic response via high-affinity binding to the Gs pathway. However, high concentrations of catecholamines via sympathetic stimulation would saturate the Gs pathway and switch to bind to Gi pathway with low affinity [22]. The activation of the Gi pathway protects against myocardial damage caused by excessive contraction via negatively inotropic action [22]. Apical contraction is weaker than basal ventricular myocardium, which shows a similar shape as octopus pot, because of the gradient density and sensitivity of βARs [12].

Although enhanced sympathetic stimulation and excess catecholamine release play a critical role in TTS, the mechanism of atypical TTS with other regional ballooning patterns (midventricular, basal, etc.) is still unknown. The hypotheses of potential involvement of catecholamine toxicity, myocardial stunning, vasospasm, and microcirculatory dysfunction have been proposed.

A biopsy study found that catecholamine-induced myocarditis was observed in TTS, suggesting catecholamine toxicity in cardiomyocytes [23]. Catecholamine overload can induce cardiomyocyte injury via calcium overload, mitochondrial damage, and rupture of the mitochondrial respiratory chain [24, 25]. Meanwhile, an extremely high level of catecholamine can cause coronary vasospasm, which contributes to cardiac stunning [26, 27]. Ischemic cardiac stunning results in regional systolic dysfunction, which shows the atypical ballooning patterns [28]. TTS exhibits coronary microvascular dysfunction in addition to epicardial coronary vasospasm. Endomyocardial biopsies in TTS reveal apoptosis of microvascular endothelial cells [29]. In addition, adenosine can improve myocardial perfusion and ejection fraction, indirectly suggesting the role of microcirculatory dysfunction in TTS [30].

## 3. TTS in Patients with COVID-19

### 3.1 Clinical Manifestations

**Fig. 1.**
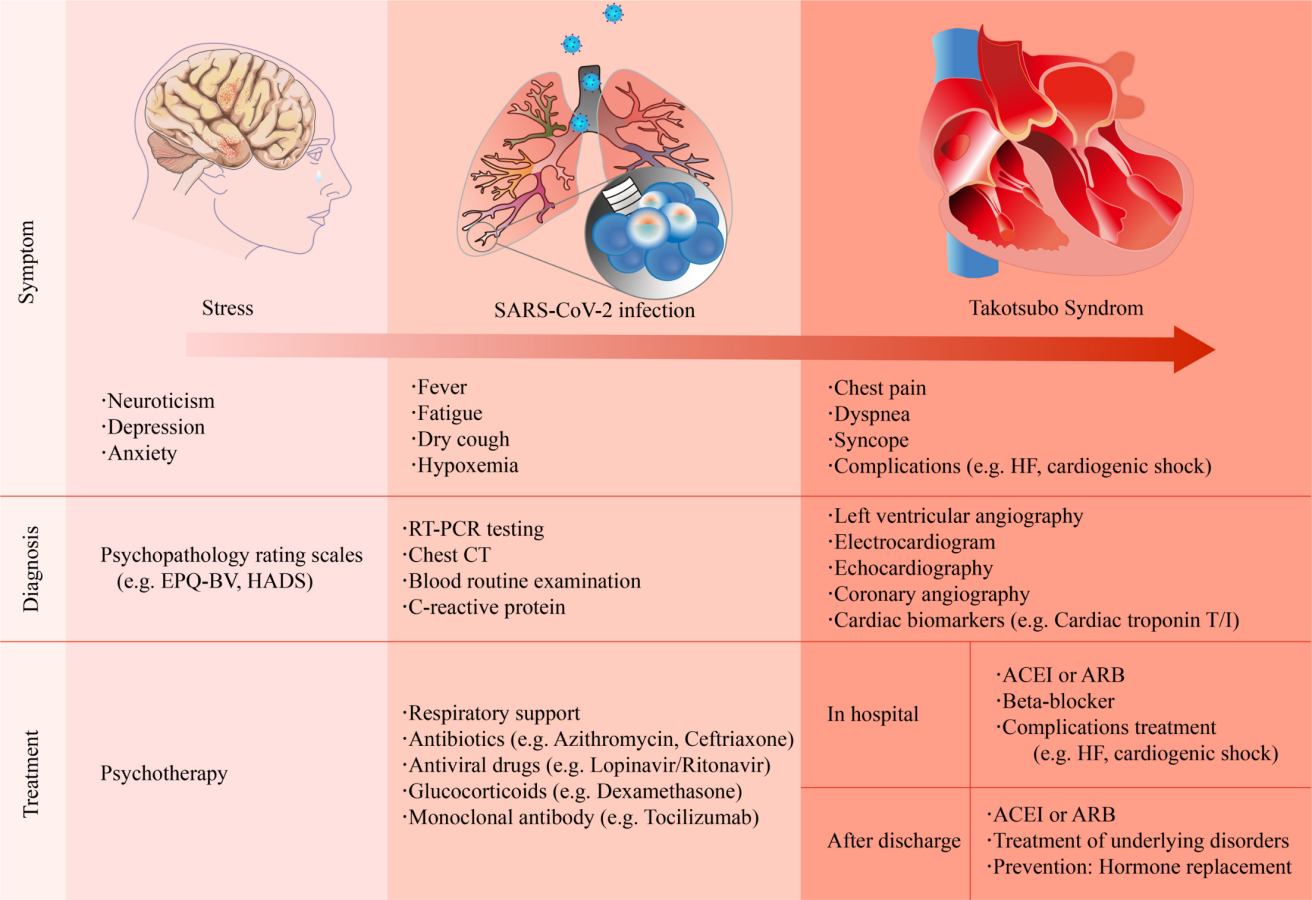
**Summary of the clinical symptoms, diagnosis, and treatment strategies of COVID-19-related Takotsubo syndrome**. ACEI, angiotensin-converting enzyme inhibitor; ARB, angiotensin II receptor blocker; CT, computed tomography; EPQ-BV, Eysenck personality questionnaire brief version; HADS, hospital anxiety and depression scale; HF, heart failure; RT-PCR, reverse transcription polymerase chain reaction.

The clinical manifestations of COVID-19-related TTS are diverse (Fig. 1). Patients with mild symptoms may experience fatigue, fever, or dyspnea [31, 32], whereas some patients present with acute chest pain [4, 33]. Patients may present with tachycardia or even cardiogenic shock when the condition deteriorates [34]. The possible triggers of TTS usually resemble those of physical stress, such as intubation and mechanical ventilation, or emotional stress, including a relative’s death because of the COVID-19 pandemic.

### 3.2 Diagnostic Investigations

The diagnosis of TTS is still a challenge because of the lack of specificity in clinical manifestations, electrocardiogram (ECG), and biomarkers. The most common ECG abnormality in TTS patients is ST-segment elevation, T-wave inversion, QTc prolongation or both [11, 35]. Most patients exhibit elevated cardiac levels of troponin, which is similar to those with an ACS. However, the peak values are relatively lower than ACS [35]. B-type natriuretic peptide (BNP), which reflects LV dysfunction, substantially increase in patients with TTS [36]. The upregulation of other potential biomarkers, such as interleukin (IL)-6 and IL-7 or microRNA-16 and microRNA-26a, is associated with stress-related disorders [37].

For suspected myocardial infarction with ST-segment elevation on ECG, emergency coronary angiography (CAG) should be performed to rule out ACS [35]. Meanwhile, an InterTAK diagnostic score can be considered for a non-ST-segment elevation on ECG, with a score of ≤70 predicting low probability for the presence of TTS and ≥70 predicting a high probability. However, it remains to be assessed with larger cohort studies if the interTAK score also allows to predict the likelihood of TTS triggered by COVID-19 [38, 39]. TTS is more likely to occur in patients with typical ballooning patterns, normal coronary arteries, and no acute infectious myocarditis confirmed by coronary computed tomography angiography, CAG or cardiac magnetic resonance imaging [35].

Among patients with COVID-19-related TTS, cardiac troponin T/I, and BNP elevated in all patients [40]. most of which were preceded by physical or emotional stress. However, left ventriculography is the gold standard for TTS.

## 4. Pathogenesis of TTS in COVID-19

**Fig. 2.**
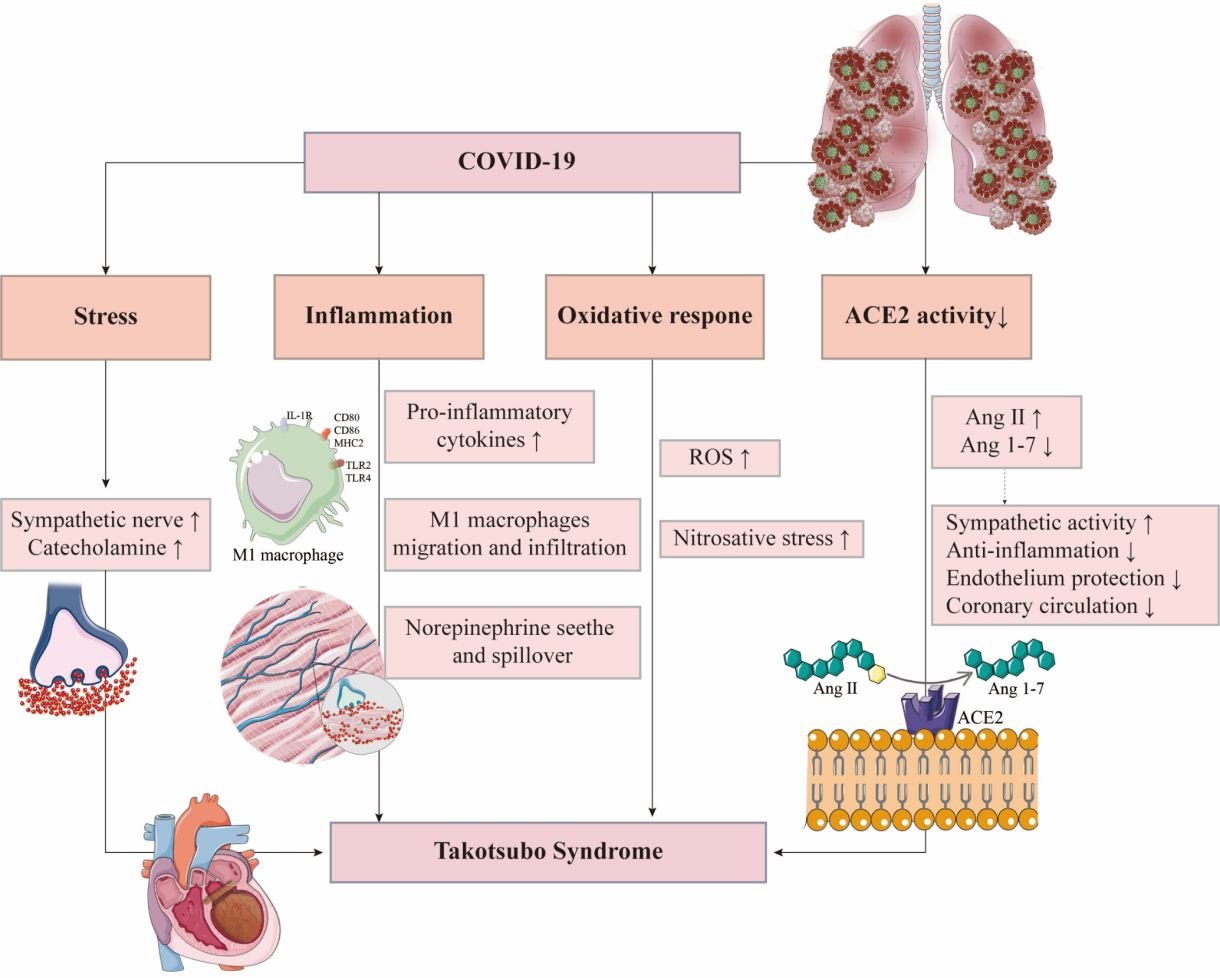
**Possible mechanisms of Takotsubo syndrome in COVID-19 patients**. ACE2, angiotensin-converting enzyme 2; Ang, angiotensin; IL-1R, interleukin-1 receptor; MHC2, major histocompatibility complex 2; ROS, reactive oxygen species; TLR, Toll-like receptor.

Until now, 68 TTS case reports in COVID-19 have been reported. More cases may be unreported given the incomplete diagnostic criteria of TTS. Therefore, exploring the pathogenesis of TTS in COVID-19 patients is of great significance (Fig. 2).

### 4.1 Acute Stress Response

Acute psychological and physiological stressors caused by COVID-19 are one of the important triggers of TTS. The COVID-19 epidemic can cause great anxiety and depression [41]. Chadha *et al*. [33] reported a case of TTS in an elderly woman without SARS-CoV-2 infection who developed anxiety because of the COVID-19 epidemic that triggered her illness. Compared with the general population, patients with COVID-19 in a closed unit not only face fatal physical complications but also social and psychological stress, which may cause greater anxiety, depression, and other psychological disorders [42]. These predisposing factors are more likely to induce TTS.

Physical stressors in COVID-19, another important trigger of TTS, should not be ignored. Pneumonia and hypoxemia induced by acute respiratory distress syndrome (ARDS) can result in enhanced sympathetic stimulation via the carotid and aortic body receptors [43]. In addition, strong acute systemic inflammatory response leads to decreased vascular resistance, followed by the sympathetic activation to ensure a sufficient blood supply for vital organs, which would be an important trigger for TTS [44].

### 4.2 Inflammation

SARS-CoV-2 causes ARDS by infecting the alveolar epithelial cells, resulting in the release of cytokines such as IL-1, IL-6, and TNF-α, leading to the release of large amounts of proteases and pro-inflammatory cytokines that cause acute inflammatory injury [45]. Given that much less myocardial injury occurs in TTS (slight elevation of cardiac markers), CRP levels are significantly elevated and comparable with ACS [46] which reveals the role of inflammation in the pathology of the disease.

TTS is pathologically characterized by inflammatory infiltration of myocardial M1 macrophage (Mon 1) [47]. During the acute inflammatory phase, Mon 1 is recruited and accumulates in the injured myocardial areas, which aggravates inflammation by protein degradation [46]. Notably, myocardial macrophage infiltration is more likely to result from the migration of circulating monocytes into the heart, rather than the proliferation of intrinsic myocardial macrophages [48]. Plasma levels of IL-6, IL-8, and chemokine ligand 1 (CXCL1) were significantly increased in TTS patients. IL-8 and CXCL1 were related to the adhesion and infiltration of macrophages. There is evidence that IL-6 is correlated with the incidence of mortality and adverse effects of TTS [49]. The risk of adverse effects was estimated to be as high as 67% with increased levels of IL-6 and IL-10 on admission because of TTS [49]. Remarkably, cytokines such as IL-1 and TNF-α can also lead to the overactivation and disruption of the cardiac sympathetic nerve terminals, causing noradrenaline spillover, and act as another powerful triggering factor for TTS [50].

### 4.3 Oxidative Stress

SARS-CoV-2 causes pulmonary inflammatory infiltration and edema via the invasion of alveolar epithelial cells. Subsequently, alveolar gas exchange is interfered by the formation of hyaline membrane and interstitial thickening. Ventilation-perfusion abnormality results in hypoxemia, which could induce the imbalance between oxidation and antioxidation and induce oxidative stress [51]. Notably, oxidative stress plays an important role in the cardiac dysfunction in TTS. Interestingly, myocardial oxidative stress level was markedly increased in patients with TTS, which is even higher than that of acute myocardial infarction, and it was increased proportionally with wall motion score and norepinephrine level [52]. The imbalance between oxidation and antioxidation leads to reactive oxygen species accumulation, resulting in the oxidation of nitric oxide to peroxynitrite anion (${\mathrm{ONOO}}^{-}$). ${\mathrm{ONOO}}^{-}$-mediated nitrosative stress could lead to protein nitration and negative inotropy [19]. TTS patients have been reported to have marked aggravation of nitrosative stress [53]. Nitrosative stress markers are directly correlated with TTS severity and varied inversely with the extent of recovery at 3 months [54]. Similarly, in animal models, suppression of nitrification stress via hydrogen sulfide could improve myocardial oxidative damage, playing a protective role in TTS [55].

### 4.4 ACE2 

ACE2 is a SARS-CoV-2 receptor required for cell entry. With the help of TMPRSS2, ACE2 on the cell membrane could be cleaved, which plays a key role in the intracellular entry of SARS-CoV-2 [56]. TMPRSS2 enzymolysis causes ACE2 dysfunction, suggesting that SARS-CoV-2 infection would decrease ACE2 activity [57]. Meanwhile, ACE2 is a negative regulator of the renin-angiotensin system and plays an essential role in cardioprotective functions. ACE2 converts angiotensin II (Ang II) into Ang 1–7. The activation of Ang 1–7/Mas pathway and reduced Ang II would have protective effects for TTS, such as anti-inflammation, protection of vascular endothelial function, improvement of coronary microcirculation, and reduction of oxidative stress and sympathetic activity [58, 59]. Therefore, ACE2 dysfunction caused by SARS-CoV-2 infection is likely to be the predisposing factor that makes patients more susceptible to TTS.

## 5. Therapeutic Strategies

TTS has been initially considered as a benign disorder. However, the morbidity and mortality of TTS were underestimated. Tornvall *et al*. [60] reported that the mortality curves in TTS patients were similar to that in patients with ACS. Any complications, such as heart failure and cardiogenic shock, might be life-threatening. The optimal treatments for acute and chronic TTS are needed for a favorable outcome.

### 5.1 Short-Term Treatment

Very recently, an international expert consensus on TTS summarized the management for patients with TTS [35]. Pulmonary edema and cardiogenic shock are the most severe complications in patients with TTS. The presence of left ventricular outflow tract obstruction (LVOTO) in shock patients should be immediately evaluated using angiography. Fluid therapy, β-blockers, and LV-assist device should be considered for LVOTO emergency. Furthermore, as reported recently, levosimendan, a ${\mathrm{Ca}}^{2+}$ sensitizer, could be safely used as a noncatecholamine inotrope in TTS patients with primary pump failure [61]. The administration of diuretics and nitroglycerin is useful for TTS patients with heart failure or pulmonary edema to reduce ventricular pressure. Of note, because of the hemodynamic instability and hypoxemia from cardiogenic shock, extracorporeal membrane oxygenation could be used as a lifesaving alternative [62]. In addition, the uncoordinated ventricular contraction may entail a thrombotic risk. Anticoagulation or antiplatelet therapy is recommended in these patients until the recovery of LVEF [35, 63].

COVID-19-related TTS is widely reported with the increasing number of confirmed COVID-19 cases. This syndrome automatically recovers when pneumonia subsides (**Supplementary Material 1**). Monoclonal antibody, such as tocilizumab, may help reduce the excessive release of catecholamines caused by the cytokine storm, which may be beneficial to reduce physical stress [64]. However, the adverse effects of drug interactions between treatments of COVID-19 and TTS are worth mentioning. A joint statement issued by the American College of Cardiology, the American Heart Association, and the American Heart Rhythm Society urgently pointed out that anti-COVID-19 drugs, such as hydroxychloroquine and azithromycin, may prolong QT intervals or lead to the occurrence of torsades de pointes-type ventricular tachycardia [65].

### 5.2 Long-Term Treatment

For patients with relatively stable TTS, ACEI, β-blockers, and diuretics can be used as preventive treatments. Nevertheless, using β-blockers and ACEI in patients with chronic TTS is reasonable; however, currently, there is insufficient clinical evidence to prevent recurrence [66].

SARS-CoV-2 invasion leads to the downregulation of surface ACE2 expression [67], while ACEI, angiotensin receptor blockers, and mineralocorticoid receptor antagonist will increase ACE2 expression [67]. However, given the lack of data on the link between the downregulation of ACE2 and patients’ susceptibility to COVID-19, these drugs should remain for patients with cardiovascular diseases [68]. Meanwhile, considering the uncertainty, calcium channel blockers, if necessary, could be taken into consideration.

TTS is prevalent in menopausal women. The role of estrogen has also been emphasized. Experimental studies reported that a weaker estrogen signaling leads to increased morbidity and mortality in both male and female mice infected with respiratory virus [69]. However, currently, there are few clinical trials to support this therapy.

## 6. Conclusions

A total of 119 patients of COVID-19-related TTS have been reported. This acute and reversible cardiac syndrome may be directly caused by SARS-CoV-2, the massive release of catecholamine triggered by hypoxemia, cytokine storm, or extreme anxiety caused by the COVID-19 pandemic. The increase of myocardial biomarkers is a sensitive warning of COVID-19-related cardiac injury. Patients with acute chest pain, ECG abnormality, and myocardial biomarkers should be suspected of cardiac complications. Echocardiography or LV angiography could help confirm the diagnosis. The portable echocardiography should be promptly used for patients with urgent medical conditions to identify LVOTO. Emergent treatment for cardiogenic shock should be implemented promptly, including mechanical ventilation or circulatory support devices. Medications, particularly QT-prolonging drugs, must be taken cautiously for TTS patients with COVID-19.

## Abbreviations

ACE2, angiotensin-converting enzyme 2; ACS, acute coronary syndrome; AF, atrial fibrillation; Ang II, angiotensin II; ARDS, acute respiratory distress syndrome; βARs, β-adrenergic receptors; BNP, brain natriuretic peptide; CAD, coronary artery disease; CAG, coronary angiography; CKD, chornic kidney disease; CK-MB, creatine kinase-MB; CMRI, cardiac magnetic resonance imaging; COPD, chronic obstructive pulmonary disease; COVID-19, coronavirus disease 2019; cTnT, cardiac troponin T; CXCL1, chemokine ligand 1; DM, diabetes mellitus; ECG, electrocardiograph; ECMO, extracorporeal membrane oxygenation; EF, ejection fraction; EMB, endocardial biopsy; F, female; HTN, hypertension; IL, interleukin; LAD, left anterior descending; LVA, left ventricular angiography; LV, left ventricular; LVOTO, left ventricular outflow tract obstruction; EF, ejection fraction; M, male; Mon 1, M1 macrophage; NT-proBNP, N-terminal pro brain natriuretic peptide; ${\mathrm{ONOO}}^{-}$, peroxynitrite anion; SARS-CoV-2, severe acute respiratory syndrome coronavirus 2; TTE, transthoracic echocardiography; TTS, Takotsubo syndrome.

## Supplementary Material

Supplementary material associated with this article can be found, in the online version, at https://doi.org/10.31083/j.rcm2309298.

## References

[1] Clerkin KJ, Fried JA, Raikhelkar J, Sayer G, Griffin JM, Masoumi A (2020). COVID-19 and Cardiovascular Disease. *Circulation*.

[2] Liu PP, Blet A, Smyth D, Li H (2020). The Science Underlying COVID-19: Implications for the Cardiovascular System. *Circulation*.

[3] Shi S, Qin M, Shen B, Cai Y, Liu T, Yang F (2020). Association of Cardiac Injury with Mortality in Hospitalized Patients with COVID-19 in Wuhan, China. *JAMA Cardiology*.

[4] Meyer P, Degrauwe S, Van Delden C, Ghadri J, Templin C (2020). Typical takotsubo syndrome triggered by SARS-CoV-2 infection. *European Heart Journal*.

[5] Pelliccia F, Pasceri V, Patti G, Tanzilli G, Speciale G, Gaudio C (2019). Long-Term Prognosis and Outcome Predictors in Takotsubo Syndrome: A Systematic Review and Meta-Regression Study. *JACC: Heart Failure*.

[6] Akashi YJ, Nef HM, Lyon AR (2015). Epidemiology and pathophysiology of Takotsubo syndrome. *Nature Reviews Cardiology*.

[7] Dote K, Sato H, Tateishi H, Uchida T, Ishihara M (1991). Myocardial stunning due to simultaneous multivessel coronary spasms: a review of 5 cases. *Journal of Cardiology*.

[8] Minhas AS, Hughey AB, Kolias TJ (2015). Nationwide Trends in Reported Incidence of Takotsubo Cardiomyopathy from 2006 to 2012. *The American Journal of Cardiology*.

[9] Lyon AR, Bossone E, Schneider B, Sechtem U, Citro R, Underwood SR (2016). Current state of knowledge on Takotsubo syndrome: a Position Statement from the Taskforce on Takotsubo Syndrome of the Heart Failure Association of the European Society of Cardiology. *European Journal of Heart Failure*.

[10] Ghadri JR, Sarcon A, Diekmann J, Bataiosu DR, Cammann VL, Jurisic S (2016). Happy heart syndrome: role of positive emotional stress in takotsubo syndrome. *European Heart Journal*.

[11] Templin C, Ghadri JR, Diekmann J, Napp LC, Bataiosu DR, Jaguszewski M (2015). Clinical Features and Outcomes of Takotsubo (Stress) Cardiomyopathy. *The New England Journal of Medicine*.

[12] Kato K, Lyon AR, Ghadri J, Templin C (2017). Takotsubo syndrome: aetiology, presentation and treatment. *Heart*.

[13] Mausbach BT, Dimsdale JE, Ziegler MG, Mills PJ, Ancoli-Israel S, Patterson TL (2005). Depressive Symptoms Predict Norepinephrine Response to a Psychological Stressor Task in Alzheimer’s Caregivers. *Psychosomatic Medicine*.

[14] Hiestand T, Hänggi J, Klein C, Topka MS, Jaguszewski M, Ghadri JR (2018). Takotsubo Syndrome Associated with Structural Brain Alterations of the Limbic System. *Journal of the American College of Cardiology*.

[15] Vriz O, Minisini R, Citro R, Guerra V, Zito C, De Luca G (2011). Analysis of beta1 and beta2-adrenergic receptors polymorphism in patients with apical ballooning cardiomyopathy. *Acta Cardiologica*.

[16] Cocco G, Chu D (2007). Stress-induced cardiomyopathy: a review. *European Journal of Internal Medicine*.

[17] El Mahmoud R, Mansencal N, Pilliére R, Leyer F, Abbou N, Michaud P (2008). Prevalence and characteristics of left ventricular outflow tract obstruction in Tako-Tsubo syndrome. *American Heart Journal*.

[18] Ueyama T, Kasamatsu K, Hano T, Tsuruo Y, Ishikura F (2008). Catecholamines and Estrogen are Involved in the Pathogenesis of Emotional Stress-induced Acute Heart Attack. *Annals of the New York Academy of Sciences*.

[19] Ghadri J, Wittstein IS, Prasad A, Sharkey S, Dote K, Akashi YJ (2018). International Expert Consensus Document on Takotsubo Syndrome (Part i): Clinical Characteristics, Diagnostic Criteria, and Pathophysiology. *European Heart Journal*.

[20] Kastaun S, Gerriets T, Tschernatsch M, Yeniguen M, Juenemann M (2016). Psychosocial and psychoneuroendocrinal aspects of Takotsubo syndrome. *Nature Reviews Cardiology*.

[21] Kawano H, Okada R, Yano K (2003). Histological study on the distribution of autonomic nerves in the human heart. *Heart and Vessels*.

[22] Paur H, Wright PT, Sikkel MB, Tranter MH, Mansfield C, O’Gara P (2012). High levels of circulating epinephrine trigger apical cardiodepression in a beta2-adrenergic receptor/Gi-dependent manner: a new model of Takotsubo cardiomyopathy. *Circulation*.

[23] Wittstein IS (2012). Stress Cardiomyopathy: a Syndrome of Catecholamine-Mediated Myocardial Stunning. *Cellular and Molecular Neurobiology*.

[24] Nef HM, Mollmann H, Troidl C, Kostin S, Voss S, Hilpert P (2009). Abnormalities in intracellular Ca2+ regulation contribute to the pathomechanism of Tako-Tsubo cardiomyopathy. *European Heart Journal*.

[25] Ellison GM, Torella D, Karakikes I, Purushothaman S, Curcio A, Gasparri C (2007). Acute β-Adrenergic Overload Produces Myocyte Damage through Calcium Leakage from the Ryanodine Receptor 2 but Spares Cardiac Stem Cells. *Journal of Biological Chemistry*.

[26] Yoshida T, Hibino T, Kako N, Murai S, Oguri M, Kato K (2007). A pathophysiologic study of tako-tsubo cardiomyopathy with F-18 fluorodeoxyglucose positron emission tomography. *European Heart Journal*.

[27] Chen W, Dilsizian V (2017). Exploring the Pathophysiology of Takotsubo Cardiomyopathy. *Current Cardiology Reports*.

[28] Sanchez-Recalde A, Costero O, Oliver JM, Iborra C, Ruiz E, Sobrino JA (2006). Pheochromocytoma-Related Cardiomyopathy: inverted Takotsubo contractile pattern. *Circulation*.

[29] Uchida Y, Egami H, Uchida Y, Sakurai T, Kanai M, Shirai S (2010). Possible Participation of Endothelial Cell Apoptosis of Coronary Microvessels in the Genesis of Takotsubo Cardiomyopathy. *Clinical Cardiology*.

[30] Galiuto L, De Caterina AR, Porfidia A, Paraggio L, Barchetta S, Locorotondo G (2010). Reversible coronary microvascular dysfunction: a common pathogenetic mechanism in Apical Ballooning or Tako-Tsubo Syndrome. *European Heart Journal*.

[31] Qin C, Zhou L, Hu Z, Zhang S, Yang S, Tao Y (2020). Dysregulation of Immune Response in Patients with Coronavirus 2019 (COVID-19) in Wuhan, China. *Clinical Infectious Diseases*.

[32] Roca E, Lombardi C, Campana M, Vivaldi O, Bigni B, Bertozzi B (2020). Takotsubo Syndrome Associated with COVID-19. *The European Journal of Case Reports in Internal Medicine*.

[33] Chadha S (2020). ‘COVID-19 pandemic’ anxiety-induced Takotsubo cardiomyopathy. *QJM: an International Journal of Medicine*.

[34] Minhas AS, Scheel P, Garibaldi B, Liu G, Horton M, Jennings M (2020). Takotsubo Syndrome in the Setting of COVID-19. *JACC: Case Reports*.

[35] Ghadri J, Wittstein IS, Prasad A, Sharkey S, Dote K, Akashi YJ (2018). International Expert Consensus Document on Takotsubo Syndrome (Part II): Diagnostic Workup, Outcome, and Management. *European Heart Journal*.

[36] Madhavan M, Borlaug BA, Lerman A, Rihal CS, Prasad A (2009). Stress hormone and circulating biomarker profile of apical ballooning syndrome (Takotsubo cardiomyopathy): insights into the clinical significance of B-type natriuretic peptide and troponin levels. *Heart*.

[37] Jaguszewski M, Osipova J, Ghadri J-, Napp LC, Widera C, Franke J (2014). A signature of circulating microRNAs differentiates takotsubo cardiomyopathy from acute myocardial infarction. *European Heart Journal*.

[38] Reper PA, Oguz F, Henrie J, Horlait G (2020). Takotsubo syndrome associated with Covid 19: and the interTAK diagnosis score. *JACC: Case Reports*.

[39] Minhas AS, Hays AG (2020). Reply: Takotsubo Syndrome Associated With COVID 19: And the InterTAK Diagnosis Score. *JACC: Case Reports*.

[40] Moderato L, Monello A, Lazzeroni D, Binno S, Giacalone R, Ferraro S (2020). Takotsubo syndrome during SARS-CoV-2 pneumonia: a possible cardiovascular complication. *Giornale Italiano di Cardiologia (Rome)*.

[41] Wang C, Pan R, Wan X, Tan Y, Xu L, Ho CS (2020). Immediate Psychological Responses and Associated Factors during the Initial Stage of the 2019 Coronavirus Disease (COVID-19) Epidemic among the General Population in China. *International Journal of Environmental Research and Public Health*.

[42] Kim S, Su K (2020). Using psychoneuroimmunity against COVID-19. *Brain, Behavior, and Immunity*.

[43] Semenza GL, Prabhakar NR (2018). The role of hypoxia-inducible factors in carotid body (patho) physiology. *The Journal of Physiology*.

[44] Balk RA (2014). Systemic inflammatory response syndrome (SIRS): where did it come from and is it still relevant today. *Virulence*.

[45] Bernard GR, Artigas A, Brigham KL, Carlet J, Falke K, Hudson L (1994). The American-European Consensus Conference on ARDS. Definitions, mechanisms, relevant outcomes, and clinical trial coordination. *American Journal of Respiratory and Critical Care Medicine*.

[46] Ciutac AM, Dawson D (2021). The role of inflammation in stress cardiomyopathy. *Trends in Cardiovascular Medicine*.

[47] Scally C, Abbas H, Ahearn T, Srinivasan J, Mezincescu A, Rudd A (2019). Myocardial and Systemic Inflammation in Acute Stress-Induced (Takotsubo) Cardiomyopathy. *Circulation*.

[48] Mylonas KJ, Jenkins SJ, Castellan RFP, Ruckerl D, McGregor K, Phythian-Adams AT (2015). The adult murine heart has a sparse, phagocytically active macrophage population that expands through monocyte recruitment and adopts an ‘M2’ phenotype in response to Th2 immunologic challenge. *Immunobiology*.

[49] Santoro F, Tarantino N, Ferraretti A, Ieva R, Musaico F, Guastafierro F (2016). Serum interleukin 6 and 10 levels in Takotsubo cardiomyopathy: Increased admission levels may predict adverse events at follow-up. *Atherosclerosis*.

[50] Y-Hassan S, Settergren M, Henareh L (2014). Sepsis-induced myocardial depression and takotsubo syndrome. *Acute Cardiac Care*.

[51] Krylatov AV, Maslov LN, Voronkov NS, Boshchenko AA, Popov SV, Gomez L (2018). Reactive Oxygen Species as Intracellular Signaling Molecules in the Cardiovascular System. *Current Cardiology Reviews*.

[52] Takuma N, Shigeki K, Seiko O, Hironori I, Takeki M, Yosuke M (2015). A Marked Increase in Myocardial Oxidative Stress Associated With Sympathetic Hyperactivity is Related to Transient Myocardial Dysfunction in Patients With Takotsubo Cardiomyopathy. *Circulation*.

[53] Surikow SY, Nguyen TH, Stafford I, Chapman M, Chacko S, Singh K (2018). Nitrosative Stress as a Modulator of Inflammatory Change in a Model of Takotsubo Syndrome. *JACC: Basic to Translational Science*.

[54] Nguyen TH, Neil CJ, Sverdlov AL, Ngo DT, Chan WP, Heresztyn T (2013). Enhanced no Signaling in Patients with Takotsubo Cardiomyopathy: Short-Term Pain, Long-Term Gain. *Cardiovascular Drugs and Therapy*.

[55] Zhang Z, Jin S, Teng X, Duan X, Chen Y, Wu Y (2017). Hydrogen sulfide attenuates cardiac injury in takotsubo cardiomyopathy by alleviating oxidative stress. *Nitric Oxide*.

[56] Hoffmann M, Kleine-Weber H, Schroeder S, Krüger N, Herrler T, Erichsen S (2020). SARS-CoV-2 Cell Entry Depends on ACE2 and TMPRSS2 and is Blocked by a Clinically Proven Protease Inhibitor. *Cell*.

[57] Xiao L, Sakagami H, Miwa N (2020). ACE2: The key Molecule for Understanding the Pathophysiology of Severe and Critical Conditions of COVID-19: Demon or Angel. *Viruses*.

[58] Simões e Silva A, Silveira K, Ferreira A, Teixeira M (2013). ACE2, angiotensin-(1-7) and Mas receptor axis in inflammation and fibrosis. *British Journal of Pharmacology*.

[59] Xia H, Suda S, Bindom S, Feng Y, Gurley SB, Seth D (2011). ACE2-mediated reduction of oxidative stress in the central nervous system is associated with improvement of autonomic function. *PLoS ONE*.

[60] Tornvall P, Collste O, Ehrenborg E, Järnbert-Petterson H (2016). A Case-Control Study of Risk Markers and Mortality in Takotsubo Stress Cardiomyopathy. *Journal of the American College of Cardiology*.

[61] Santoro F, Ieva R, Ferraretti A, Ienco V, Carpagnano G, Lodispoto M (2013). Safety and Feasibility of Levosimendan Administration in Takotsubo Cardiomyopathy: a Case Series. *Cardiovascular Therapeutics*.

[62] Chow J, Alhussaini A, Calvillo-Argüelles O, Billia F, Luk A (2020). Cardiovascular Collapse in COVID-19 Infection: The Role of Veno-Arterial Extracorporeal Membrane Oxygenation (VA-ECMO). *CJC Open*.

[63] Santoro F, Stiermaier T, Tarantino N, De Gennaro L, Moeller C, Guastafierro F (2017). Left Ventricular Thrombi in Takotsubo Syndrome: Incidence, Predictors, and Management: Results From the GEIST (German Italian Stress Cardiomyopathy) Registry. *Journal of the American Heart Association*.

[64] Luo P, Liu Y, Qiu L, Liu X, Liu D, Li J (2020). Tocilizumab treatment in COVID‐19: a single center experience. *Journal of Medical Virology*.

[65] Roden DM, Harrington RA, Poppas A, Russo AM (2020). Considerations for Drug Interactions on QTc in Exploratory COVID-19 Treatment. *Circulation*.

[66] Isogai T, Matsui H, Tanaka H, Fushimi K, Yasunaga H (2016). Early beta-blocker use and in-hospital mortality in patients with Takotsubo cardiomyopathy. *Heart*.

[67] Gheblawi M, Wang K, Viveiros A, Nguyen Q, Zhong J, Turner AJ (2020). Angiotensin-Converting Enzyme 2: SARS-CoV-2 Receptor and Regulator of the Renin-Angiotensin System: Celebrating the 20th Anniversary of the Discovery of ACE2. *Circulation Research*.

[68] Bozkurt B, Kovacs R, Harrington B (2020). Joint HFSA/ACC/AHA Statement Addresses Concerns re: Using RAAS Antagonists in COVID-19. *Journal of Cardiac Failure*.

[69] Suba Z (2020). Prevention and Therapy of COVID-19 via Exogenous Estrogen Treatment for Both Male and Female Patients; An Opinion Paper. *Journal of Pharmaceutical Sciences*.

